# New Stability-Indicating RP-UFLC Method for Determination of Trospium Chloride in Tablet Dosage Form

**DOI:** 10.3797/scipharm.1207-07

**Published:** 2012-08-31

**Authors:** Sagar Suman Panda, Bera V. V. Ravi Kumar, Ganeswar Mohanta, Rabisankar Dash, Pinkal Kumar Patel

**Affiliations:** Department of Pharmaceutical Analysis and Quality Assurance, Roland Institute of Pharmaceutical Sciences, Khodasingi, 760010, Berhampur (Odisha), India.

**Keywords:** Trospium chloride, UFLC, Forced degradation, TBAHS

## Abstract

A simple, precise, and accurate isocratic RP-UFLC stability-indicating assay method has been developed to determine trospium chloride in tablet dosage form. Isocratic separation was achieved on an Enable-C18G (250 mm × 4.6 mm i.d., particle size 5 μm) column at room temperature, the mobile phase consisted of acetonitrile:0.01M TBAHS (50:50, v/v) at a flow rate of 1.0 ml/min, the injection volume was 20 μl, and PDA detection was carried out at 215 nm. The drug was subjected to acid and alkali hydrolysis, oxidation, photolysis, and heat as stress conditions. The method was validated for specificity, linearity, precision, accuracy, robustness, and system suitability. The method was linear in the drug concentration range of 10–300 μg/ml with the correlation coefficient being 0.999. The RSD for repeatability and intermediate precision was well below 2%. The mean recoveries were between 100.52–101.68% for trospium chloride.

## Introduction

**Fig. 1.**
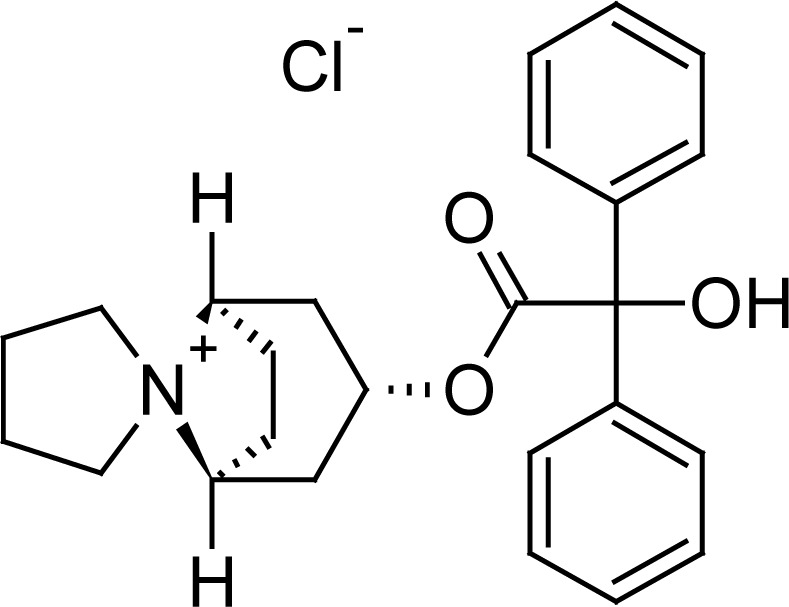
Chemical structure of Trospium chloride

Trospium chloride (TRC) is a quarternary ammonium compound used in overactive bladder management [1]. It is believed to be more effective than other anticholinergic drugs for management of the overactive bladder condition [2]. Chemically, it is 3α-benziloyloxynortropane-8-spiro-1′-pyrrolidinium chloride (Fig. 1) [3].

As per literature survey, there are very few analytical methods reported for the analysis of trospium chloride. It includes estimation of trospium in tablets by the UV spectro-photometric method, HPLC -fluorescence analysis of trospium by derivatization with benoxaprofen chloride, HPLC analysis of trospium chloride in tablets, and the LC-MS and LC-MS/MS methods for estimation of trospium in human plasma samples [4–9]. But no stability-indicating RP-UFLC method was reported until the literature survey for the determination of trospium chloride in pharmaceutical dosage form. The UFLC is a better alternative to conventional HPLC, as it decreases the analysis time and requirement of the mobile phase used in drug analysis. UFLC has been used as a time efficient and cost effective analytical tool in the estimation of a combination of drugs in combined pharmaceutical products, estimation of hallucinogenic agents in drug products, determination of the drug in microdialysis samples, and in the determination of drugs in skin diffusate samples [10–13]. So, an attempt was made to develop and validate a novel, rapid, simple, precise, and accurate RP-UFLC method for the determination of trospium chloride in tablet dosage form. Stability parameters for the drug were assessed by subjecting it to forced degradation conditions like acid-alkali hydrolysis, oxidation, thermal, and exposure to UV radiation. The developed method was also validated as per ICH guidelines for specificity, linearity, precision, accuracy, robustness, stability, and system suitability [14].

## Experimental

### Chemicals and Reagents

Analytical Grade TRC (purity > 99%) was procured from Zydus Healthcare, India. Acetonitrile (Merck Ltd., Mumbai, India) was of HPLC grade. Analytical grade sodium hydroxide, hydrochloric acid, and hydrogen peroxide were procured from S.D. Fine Chem. Ltd., Mumbai, India. Tetra butyl ammonium hydrogen sulfate (TBAHS) purchased from Hi-Media Laboratories Pvt. Ltd., Mumbai, India was of AR grade. The water for the HPLC was obtained by using the TKA Water Purification System, Germany. The marketed tablet formulation containing 10 mg of TRC was purchased from the local market.

### UFLC Instrumentation and chromatographic conditions

Quantitative UFLC was performed on a binary gradient UFLC with two Shimadzu Prominence UFLC LC-20AD pumps, with a 20μl sample injection loop (manual) and a SPD M20A PDA detector. The output signal was monitored and integrated using Shimadzu LC Solution Software. An Enable C18G column (250 mm × 4.6 mm i.d., 5 μm) was used for separation. Chromatographic analysis was carried out at ambient temperature on the column using the acetonitrile: 0.01M TBAHS (50:50, v/v) as the mobile phase at a flow rate of 1.0 ml/min in isocratic mode. The 0.01M TBAHS solution was prepared by accurately weighing 3.3954gm of TBAHS salt and dissolving it in 1000ml of HPLC grade water. Afterwards, both the acetonitrile and TBAHS were ultrasonicated (Enertech, India) up to 20 min for degassing prior to use. The PDA detection was set at 215 nm. The water bath (Thermolab, India) and UV Chamber (Jain Scientific Glass Works, Ambala, India) were used for the forced degradation study of the drugs. The analytical balance, Model-GR-202 (AND Instrument India Pvt. Ltd., Gurgaon, India) of sensitivity 0.1mg was used to weigh the chemicals and reagents.

### Preparation of Standard and Sample Solution

The standard stock solution of TRC was prepared by transferring 25mg of the drug into a 25ml volumetric flask having 10ml of the mobile phase and was ultrasonicated for 5 min. Finally, the volume was made up to the 25ml mark with the mobile phase, which gave 1000μg/ml of solution.

Twenty tablets were weighed accurately and powdered finely. A quantity of tablet powder equivalent to 25 mg of TRC was transferred into a 25 ml volumetric flask, containing 10 ml of the mobile phase, and ultrasonicated for 20 min; the volume was made up and mixed well. The solution was filtered through a 0.2μm filter to remove particulate matter, if any. The filtered solution was appropriately diluted with the mobile phase for analysis as already described. The amount of drug present in the sample solution was calculated by using the calibration curve. All of the solutions were stored at 2–8°C for future use.

### Method validation

#### Specificity

The specificity of the method was determined by checking the interference of any of the possible degradation products generated during the forced degradation study of TRC. The forced degradation of the drug was carried out with 0.1M HCl, 0.001M NaOH, 1% v/v H_2_O_2_, thermal (50°C), and photolysis (365nm) for determining the stability nature of the drug. The degraded samples were prepared by taking suitable aliquots of the drug solution, and then undertaking the respective stress testing procedures for each solution. After the fixed time period, the stressed test solutions were diluted with the mobile phase. For every stress condition, a solution of concentration 100 μg/ml of TRC was prepared. The specific stress conditions are described as follows.

Acidic degradation condition
  Acidic degradation was carried out by adding 1 ml of 0.1M HCl, and after 45 min the mixture was neutralized by adding 0.1M NaOH.
Alkali degradation condition
  Alkali degradation was carried out by adding 1 ml of 0.001M NaOH, and after 45 min the mixture was neutralized by adding 0.001M HCl.
Oxidative degradation condition
  Oxidative degradation was performed by exposing the drug to 1 ml of 1% (v/v) H_2_O_2_ for 45 min.
Thermal degradation condition
  Thermal degradation was performed by heating the drug content at 50°C on a thermostatically controlled water bath for 45 min.
Photolytic degradation condition
  Photolytic degradation was carried out by exposing the drug content to UV light (365nm) inside a UV chamber for 180 min.

#### Linearity

An eight point calibration curve was obtained over a concentration range of 10 to 300 μg /ml for TRC. The calibration curve was plotted by taking the average peak area (n=3) on the y-axis and concentration (μg /ml) on the x-axis.

#### Precision

The repeatability (intra-day precision) of the method was ascertained from the peak areas obtained by the actual determination of six replicates of a fixed amount of drug. For intermediate precision (inter-day precision) of the method, the same procedure described above was carried out by a different analyst on a different day under similar experimental conditions. The percent RSD values were calculated for both types of the precision study.

#### Accuracy

To check the accuracy of the proposed method, recovery studies were carried out at 80,100, and 120% of the test concentrations. The recovery study was performed three times at each level. The amount of TRC present in the sample was calculated using the calibration curve.

#### Robustness

The robustness of the method was studied by deliberately changing method parameters like flow rate of the mobile phase, detection wavelength, and organic phase composition. A series of system suitability parameters like retention time, theoretical plates, and tailing factor were determined for each modified condition as per ICH [14]. Solution stability of the drug in the mobile phase was determined by keeping the drug solution at ambient conditions for 24h.

#### Limit of detection and Limit of quantitation

The LOD (limit of detection) and LOQ (limit of quantitation) were determined based on the 3.3 and 10 times the standard deviation of the response, respectively, divided by the slope of the calibration curve.

## Results and Discussion

### Optimization of the Chromatographic conditions

**Fig. 2.**
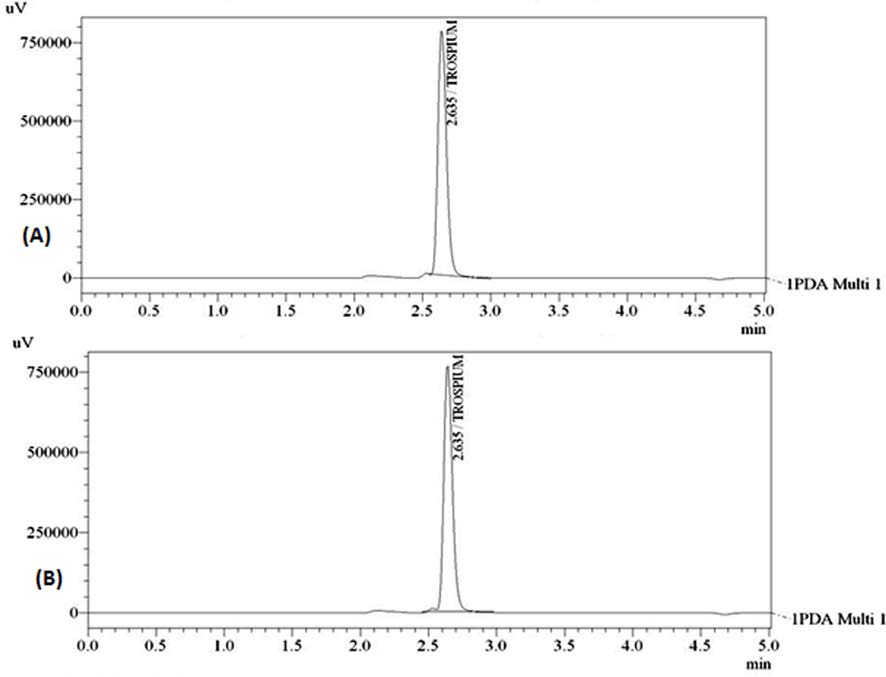
Chromatograms of TRC (A) standard drug, (B) tablet dosage form

Optimization of the mobile phase was carried out based on the tailing factor and theoretical plates obtained for TRC. During the trial runs, different mobile phase compositions like methanol:water, methanol:0.01M TBAHS, acetonitrile:water, acetonitrile:0.01M TBAHS, at various compositions (50:50,60:40,70:30,75:25;v/v), and flow rates (0.8,1.0 and 1.2ml/min) were tested for selection. The mobile phase consisting of acetonitrile: 0.01M TBAHS (50:50, v/v) at a flow rate of 1.0ml/min was selected, which gave sharp, symmetric peaks having a tailing factor of 1.36. The retention time for TRC was found to be 2.635min. The run time was set at 5min for time-efficient analysis of the drug. The theoretical plate count was found to be 6722. The PDA detection was set at 215nm. The separation was carried out at room temperature. Fig. 2 (A) and (B) represent the chromatograms of the TRC standard drug and marketed tablet dosage form, respectively.

### Specificity

**Fig. 3.**
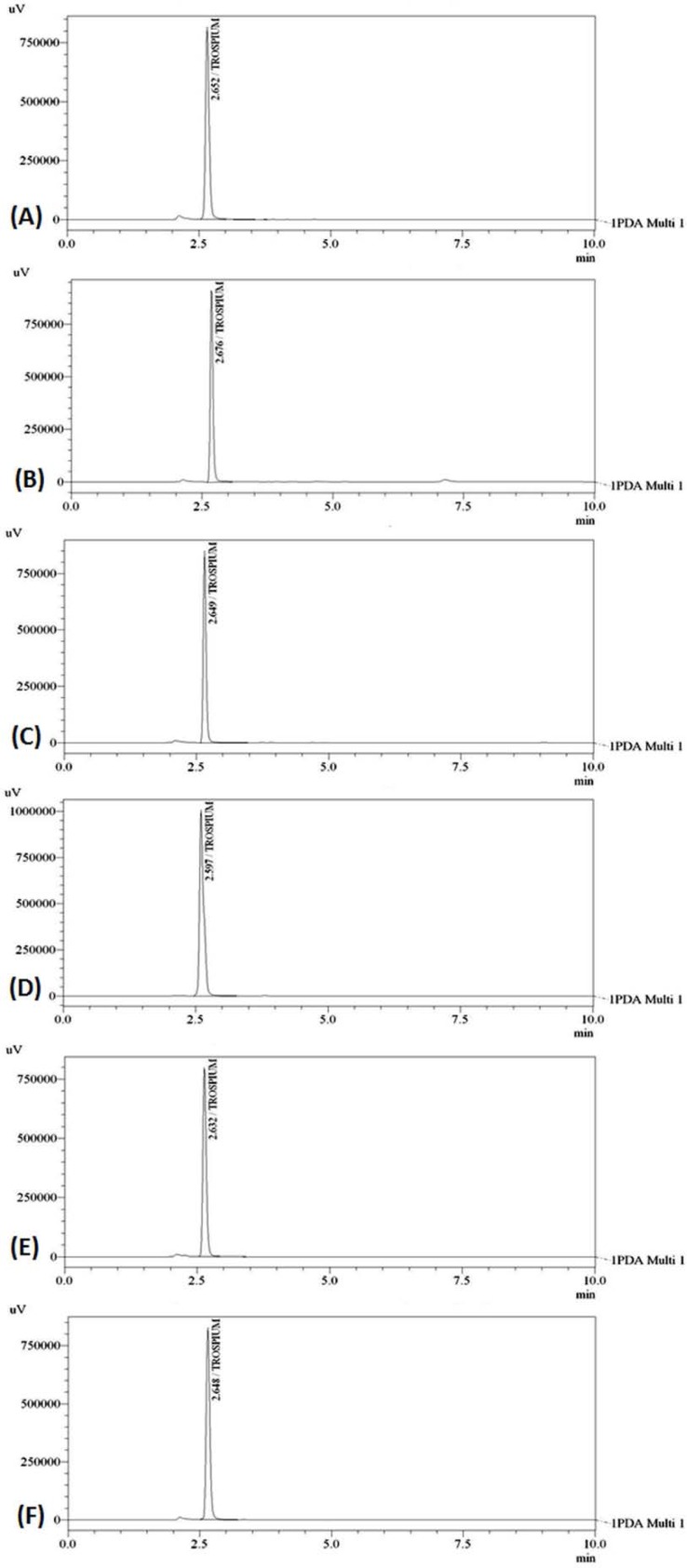
Chromatograms of TRC 100μg/ml (A) untreated drug, (B) acid-degraded drug, (C) alkali-degraded drug, (D) oxidation-degraded drug, (E) thermal-degraded drug and (F), photolysis-degraded drug

To evaluate specificity, the PDA detector was applied to determine peak purity of the chromatographic peaks obtained for the stress treated drug solution. The peak purity results are indicative for determining the peak homogeneity. TRC undergoes complete degradation under the alkaline stress condition by using 0.1M NaOH and thermal stress conditions by heating at 80°C in a thermostatically controlled water bath. So, the stress conditions were optimized in order to obtain degradation of TRC within 10–20% of the amount of drug taken. The modified alkaline stress was applied by using 0.001M of NaOH solution. In the case of thermal degradation, the applied temperature was decreased to 50°C. TRC shows moderate degradation in the applied acidic, alkaline, and thermal stress conditions, and was found to be stable under oxidation and photolysis stress conditions. Fig. 3 (A), (B), (C),(D),(E), and (F) represents the chromatograms of drug degradation of untreated drug, and degradation by acid, alkali, oxidation, thermal, and photolysis. The run time for each stressed drug solution was increased from 5 min to 10 min in order to find the presence of any extra peak due to the possible degradation of TRC. But no such extra peaks were found in the chromatogram. Also, the obtained peak purity values (>0.999) indicated that there was no co-eluting or hidden peaks along with the drug peak, which showed specificity and the stability-indicating nature of the method. The results of the forced degradation study are summarized in Table 1.

#### Linearity

The calibration curve was found to be linear over a concentration range of 10–300 μg/ml for TRC. The linear regression equation was *y* = 32711 x – 8092 with a correlation coefficient of 0.999.

#### Precision

The method was found to be precise as the % RSD values for repeatability and intermediate precision studies were well below 2%, confirming that the method was precise. The results are shown in Table 2.

#### Accuracy

The accuracy of the method was determined by the recoveries of TRC by standard addition methods. The values show high levels of accuracy of the method. The result of the accuracy study is shown in Table 3.

### Robustness

The method was found to be robust in accordance with deliberate changes in the mobile phase flow rate (±0.1ml/min), detection wavelength (±5nm), and the organic phase composition (±2%). The obtained results for the robustness study are shown in Table 4. The solution stability of TRC was satisfactory with a recovery of 99.16% after 24 h, indicating no significant degradation of the analyte in the selected mobile phase.

### Limit of detection and Limit of quantitation

The limit of detection (LOD) and limit of quantitation (LOQ) values were found to be 2.54μg /ml and 7.71μg/ml, respectively.

### Analysis of commercial tablet dosage form

The developed method was applied for the determination of TRC in tablet dosage form. The result of the assay (n=3) for the drug yielded 101.46% (SD = ±0.058) of TRC from the tablet dosage form. The higher percentage of recovery and non-interference of the formulation excipients in retention time of the drug shows the selectivity of the method for the estimation of TRC in tablet dosage form.

## Conclusion

A novel, validated stability-indicating RP-UFLC method has been developed for the determination of trospium chloride (TRC) in tablet dosage form. The validation study showed that the method was specific, linear, precise, accurate, and sensitive in the proposed working range. The use of an UFLC resulted in time efficient rapid analysis of trospium. The method was found to be robust with respect to the deliberate changes made in flow rate, detection wavelength, and organic phase composition. Due to these features, the method is stability-indicating in nature. The method was successfully applied for the determination of trospium chloride in tablet dosage form. Further, the developed RP-UFLC method can be applied for routine analysis of trospium chloride in API, pharmaceutical formulations, dissolution medium, and biological fluids.

## Figures and Tables

**Tab. 1.** Results of the forced degradation study

**Stress Applied**	**Retention Time (min)**	**Degradation (%)**	**Peak Purity**
Untreated	2.652	–	1.00000
0.1M HCl	2.676	10.59	0.99998
0.001M NaOH	2.649	8.28	1.00000
1% H_2_O_2_	2.597	No degradation	1.00000
50°C	2.632	5.55	1.00000
UV radiation at 365nm	2.648	No degradation	1.00000

**Tab. 2.** Precision of the method

**Precision Type**	**Concentration Taken (μg/ml)**	**Peak Area^a^ ± SD, (%RSD)**
Repeatability (Intra-day, n=6)	100	3232459.63 ± 20352.04, 0.63
Intermediate precision (Inter-day, n=6)	100	3250604 ± 18458.3, 0.57

a Mean of six determinations.

**Tab. 3.** Accuracy of the method

**Spiked Concentration (μg/ml)**	**Recovery^a^ (%) Pure Drug ± SD, RSD (%)**
80	101.68 ± 0.1607, 0.16
100	100.92 ± 0.1457, 0.14
120	100.52 ± 0.1514, 0.15

a Mean of three determinations.

**Tab. 4.** Robustness of the method

**Parameter**	**Retention Time (min.)**	**Theoretical Plates**	**Tailing Factor**
Flow rate (ml/min)			
0.9	2.919	6227	1.343
1.0	2.635	6722	1.364
1.1	2.391	6192	1.362
Wavelength (nm)			
210	2.635	6624	1.368
215	2.635	6722	1.364
220	2.634	6890	1.365
Acetonitrile (%)			
48	2.699	6954	1.367
50	2.635	6722	1.364
52	2.625	6452	1.361

	**Initial time**	**Final time**	**Recovery (%)**
Solution stability	0 hr	24 hr	99.16

## References

[1] Dmochowski RR, Rosenberg MT, Zinner NR, Staskin DR, Sand PK (2010). Extended-release trospium chloride improves quality of life in overactive bladder. Value Health.

[2] Staskin DR (2006). Trospium chloride: distinct among other anticholinergic agents available for the treatment of overactive bladder. Urol Clin North Am.

[3] Sweetman S (2009). Urological Drugs. Martindale-The Complete Drug Reference.

[4] Bendale AR, Prajapati SV, Narkhede SP, Narkhede SB, Jadav AG, Vidyasagar G (2011). Analytical method development and validation protocol for trospium chloride in tablet dosage form. Indo-Global J Pharm Sci.

[5] Schladitz-Keil G, Spahn H, Mutschler E (1985). Fluorimetric determination of the quarternary ammonium compound trospium and its metabolite in biological material after derivatization with benoxaprofen chloride. J Chromatogr B Biomed Sci Appl.

[6] Lakshmi MV, Rao JVLNS, Rao AL (2012). RP-HPLC estimation of trospium chloride in tablet dosage forms. E-J Chem.

[7] Xiang HI, Ding JS, Tan ZR (2007). Determination of trospium chloride in human plasma by HPLC-MS and study of the relative bioavailability of domestic and imported preparations. Chin J Hosp Pharm.

[8] Hotha KK, Bharathi DV, Kumar SS, Reddy YN, Chatki PK, Ravindranath LK, Jayaveera KN (2010). Determination of the quarternary ammonium compound trospium in human plasma by LC-MS/MS:application to a pharmacokinetic study. J Chromatogr B.

[9] Tang J, Tan ZR, Zhou YB, Ding JS (2011). High-performance liquid chromatography-tandem mass spectrometry for the determination of trospium chloride in human plasma and its application in a bioequivalence study. Anal Lett.

[10] Bandarkar FS, Khattab IS (2010). Simultaneous estimation of glibenclamide,gliclazide,and metformin hydrochloride from bulk and commercial products using a validated ultra fast liquid chromatography technique. J Liq Chromatogr Relat Technol.

[11] Min JZ, Shimizu Y, Toyo’oka T, Inagaki S, Kikura-Hanajiri R, Goda Y (2008). Simultaneous determination of 11 designated hallucinogenic phenethylamines by ultra-fast liquid chromatography with fluorescence detection. J Chromatogr B.

[12] Sun N, Wen J, Lu G, Hong Z, Fan G, Wu Y, Sheng C, Zhang W (2010). An ultra fast lc method for the determination of iodiconazole in microdialysis samples and its application in the calibration of laboratory-made linear probes. J Pharm Biomed Anal.

[13] Gannu R, Yamasani VV, Yamasani SK, Palem CR, Voruganti S, Yamsani MR (2009). Develpment of ultra fast liquid chromatographic method for simultaneous determination of nitrendipine and carvone in skin diffusate samples. J Pharm Biomed Anal.

[14] (2005). http://www.ich.org/products/guidelines/quality/quality-single/article/validation-of-analytical-procedures-text-and-methodology.html.

